# Development of a New Clay-Based Aerogel Composite from Ball Clay from Piauí, Brazil and Polysaccharides

**DOI:** 10.3390/polym15112412

**Published:** 2023-05-23

**Authors:** Wilton C. Lopes, Francisco M. Brito, Francisco E. Neto, Alyne R. Araújo, Rodolpho C. Leite, Vicente G. Freitas Viana, Edson C. Silva-Filho, Durcilene A. Silva

**Affiliations:** 1 Research Center on Biodiversity and Biotechnology, BIOTEC, Federal University of Delta of Parnaíba, UFDPar, São Sebastião Avenue, Parnaíba 64202-020, PI, Brazil; 2 Postgraduate Program in Materials Engineering, Federal Institute of Piaui (IFPI), Campus Teresina Central, Teresina 64001-270, PI, Brazil; 3 LIMAV, Interdisciplinary Laboratory of Advanced Materials, Piauí Federal University, Teresina 64049-550, PI, Brazil

**Keywords:** aerogel, ball clay, angico gum, thermal insulation, mechanical properties

## Abstract

The incorporation of polymeric components into aerogels based on clay produces a significant improvement in the physical and thermal properties of the aerogels. In this study, clay-based aerogels were produced from a ball clay by incorporation of angico gum and sodium alginate using a simple, ecologically acceptable mixing method and freeze-drying. The compression test showed a low density of spongy material. In addition, both the compressive strength and the Young’s modulus of elasticity of the aerogels showed a progression associated to the decrease in pH. The microstructural characteristics of the aerogels were investigated by X-ray diffraction (XRD) and scanning electron microscopy (SEM). The chemical structure was studied by infrared spectroscopy with Fourier transform (FTIR). The TGA curves from a non-oxidizing atmosphere indicated that the clay had a mass loss of 9% above 500 °C and that due to the presence of polysaccharides, the aerogels presented a decomposition of 20% at temperatures above 260 °C. The DSC curves of the aerogels demonstrated a displacement in higher temperatures. In conclusion, the results showed that aerogels of ball clay with the incorporation of polysaccharides, which are still minimally studied, have potential application as thermal insulation considering the mechanical and thermal results obtained.

## 1. Introduction

The first silica-based aerogels were developed in 1931 and 1932 by Kistler using the supercritical drying method. They were not well explored as there was no practical application for them and the drying method made their production unfeasible. The supercritical drying method consists of removing the liquid from the hydrogels through a supercritical fluid to obtain a stable material filled with air, which is of a size similar to the original hydrogel [1]. 

Recent studies show that the most viable drying process is lyophilization, which is considered a simple, ecologically acceptable, and efficient approach since water is used as a solvent, which significantly reduces the cost of production [2,3]. Recently, the postive mechanical and thermal properties of clay-based aerogels have attracted the attention of many researchers. Thus, numerous applications have been developed from aerogels, such as thermal insulation [4,5,6], drug administrators [2], catalysis [7], acoustic insulation [8], removal of heavy metal ions [9], and flame retardants [10,11,12].

In addition, the incorporation of polymer components into clay-based aerogels can improve the mechanical properties, especially toughness and mechanical strength [13]. Clay aerogel composites are a class of materials with a three-dimensional structure, permanent porosity, low density, low thermal conductivity, and fire retardant behavior [14]. Therefore, hybrid aerogels based on clay are considered a potential material, as clay is low cost, available, nontoxic, biodegradable, and easily produced [4].

Ball clay, also called plastic clay, is defined as a type of sedimentary clay with fine grain size, high plasticity, and lower refractoriness that contains kaolinite and has strong binding properties [15,16]. These clays have been widely used in agriculture, the ceramic industry, and as suspension agents or binders in several industries but have rarely been used in the production of aerogel. The term “ball clay” has no mineralogical meaning but originated in an ancient method of exploration in England, which consists of cutting the clay into cubes and then rounding them into balls to facilitate transport [17]. This type of clay is found in Brazil only in two regions: in São Simão in the state of São Paulo and in the northeast region, especially in Oeiras in the state of Piauí [18].

Angico gum (AG) is a macromolecular polysaccharide consisting of arabinose (67%), galactose (24%), rhamnose (2%), and glucuronic acid (7%) that is isolated from Anadenanthera colubrina var. cebil, known as “red angico”, which is found in several regions of Brazil, especially in the Cerrado biome. AG is an abundant biopolymer in nature and has a great potential in the production of materials in several areas because it is a great source of complex polysaccharides and has a wide ability to form films and gels [19,20]. 

Sodium alginate (SA) is a natural polysaccharide extracted from seaweed [20]. It is usually used in biomedical material to support cells and control drug delivery. SA is also a known flame-retardant material due to its low oxygen index value [21,22]. Due to the low viscosity of AG, which can make the aerogel fragile [19], combining it with SA can be an advantageous alternative to obtain better viscosity and hydrophilicity so it can be used without difficulty in modified materials such as hydrogels and sponges [23]. In addition, when combined, a new composite with properties different from those achieved with the individual use of polysaccharides can be developed. Another considerable advantage is that polysaccharides are renewable and biodegradable [24]. In the recent literature, there are no reports on the production of aerogels using angico gum, ball clay, or a combination of angico gum and ball clay. This study aims to develop an aerogel using angico gum, alginate, and ball clay, with water as the solvent and drying by the lyophilization method. In addition, in this work, studies of the mechanical and thermal properties of the aerogels produced were carried out.

## 2. Materials and Methods

### 2.1. Materials

Ball clay (C) was collected in a storage house in Oeiras city, in the state of Piauí, Brazil (Latitude: 7°1′14″ South, Longitude: 41°53′36″ West). The raw clay was ground and passed through a 200 mesh sieve with 75 µm spacing. After granulometric control, the clay was heated in an oven at 200 °C for 1 h to eliminate the organic material still present. AG exudate was collected in the city of Simplício Mendes, Piauí, Brazil (Latitude: 7°51′33″ South, Longitude: 41°53′36″ West). The isolation and purification of AG followed the method described by Silva et al. [19]. The species A. nadenanthera colubrina var. cebil (Griseb) Altschul has already been registered in the National System for the Management of Genetic Heritage and Associated Traditional Knowledge, with the number A52A49D. The SA was purchased from Sigma-Aldrich and used without any modification.

### 2.2. Preparation of Aerogel

**Figure 1:**
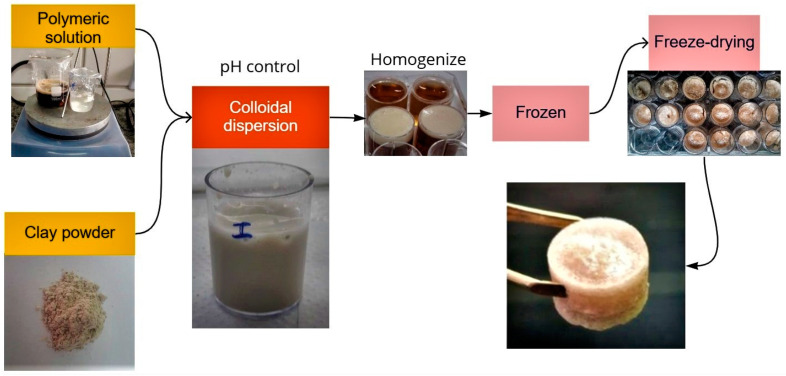
Illustration of the production process of aerogel composites based on ball clay and polysaccharides.

To prepare the aerogels, the methodology by Wang et al. [25] was employed with some adaptations. A mixed matrix, based on angico gum/alginate/clay (AG/SA/C) at a distinct AG/SA ratio and pH were used. Figure 1 shows the aerogels preparation scheme briefly. The components present in the aerogel samples are shown in Table 1. First, 50 mL of AG solution was stirred mechanically using a mini rectifier for 5 min; after that, a raw clay powder was added slowly to the solution in continuing mechanical agitation for 5 min. Then, 50 mL of SA solution was slowly added to the solution that was still in mechanical agitation for 5 min. Soon after, the pH control was made and the solution was slowly poured into plates and agitated for 5 min on a shaker table for homogenization. Finally, to obtain the aerogels, all samples were frozen at −40 °C for 48 h and subsequently placed in a freeze-dryer for another 48 h. The characteristics of the resulting samples are shown in Table 1. The numbers after AG, SA, and C are their masses in grams for every 100 mL of water and the final numbers (6, 7, and 8) indicate the pH of the solutions before being frozen.

### 2.3. Characterization

#### 2.3.1. Mechanical Performance

The compression test was performed on cylindrical samples approximately 14 mm diameter and 14 mm in height using Emic DL20000 equipment, (São José dos Pinhais, Brazil), with a 5000 N load cell. The specific modulus was determined by the ratio between the compression modulus and the apparent density.

#### 2.3.2. Scanning Electron Microscope (SEM)

The micrographs were created with a scanning electron microscope (SEM) with a field emission cannon, FEI brand, (Eindhoven, The Netherlands). Quanta FEG 250 model, with acceleration voltage of 1 to 30 kV. For the micrographs, the samples were fixed on aluminum substrate (stub) using double-sided carbon adhesive tape.

#### 2.3.3. Apparent Density

To calculate the densities of dry aerogels, an analytical balance (Mettler Toledo AB204-S), (Barueri, Brazil), was used to measure the mass. A digital caliper was used to measure the dimensions. The density for each composition was calculated from the ratio of mass by volume for each sample.

#### 2.3.4. X-ray Diffraction

##### Microstructure 

The patterns of the X-ray diffraction of ball clay, angico gum, alginate, and aerogels were investigated at room temperature at the Federal University of Piauí (UFPI) using Shimadzu equipment, (Kyoto, Japan), model XRD-6000, in normal mode with Cu-Kα radiation (λ = 1.5418), operating with a voltage of 40 kV and a current of 30 mA, and using a step of 0.02° (2θ) in the sweep range of 2θ from 5 to 75°.

##### Degree of Relative Crystallinity 

The degree of crystallinity was determined by the method used in the study performed by Zhang et al. [26]. Origin 2018 software was used in this analysis, and a smooth curve was plotted in the XRD diffractogram separating the area of the peaks, which corresponds to the crystalline portion. The area below, between the smooth curve and the rectilinear line that sweeps the 2θ range from 5 to 35°, corresponds to the amorphous part.

Equation (1) was used to calculate the degree of relative crystallinity in all samples:

(1) $$X(\%)=\frac{A_{p}}{A_{t}}\times 100$$

where *X* corresponds to the degree of relative crystallinity, *A_p_* is the area of the peaks, and *A_t_* indicates the area of the peaks plus the area below the gentle line (the amorphous area). The range of the diffractogram sweep was from 2θ = 5° to 35° in all samples.

#### 2.3.5. Fourier Transform Infrared Spectroscopy–FTIR

The chemical structure of the aerogels was verified using a Perkin Elmer infrared equipment, (São Paulo, Brazil), model Spectrum 100 with samples for KBr powder with resolution of 4 cm^−1^ in the spectral wave number range of 4000 to 400 cm^−1^ with XX accumulations.

#### 2.3.6. Thermogravimetric Analysis (TGA)

The thermogravimetric analysis was performed with a TGA-51H Shimadzu, (Kyoto, Japan), thermogravimetric analyzer. Masses of around 10 mg were used under a nitrogen flow of 50 mL/min. The heating rate was 10 °C/min between 25 °C and 800 °C. The TGA curve and DTG derivative were plotted using Origin software.

#### 2.3.7. Differential Scanning Calorimetry (DSC)

DSC was obtained with the Shimadzu, (Kyoto, Japan), model DSC-60 plus Differential Scanning Calorimeter, operating in the temperature range of 25 to 600 °C at a heating rate of 10 °C/min and in a nitrogen atmosphere with a flow rate of 50 mL/min.

## 3. Results and Discussion 

### 3.1. Mechanical Properties

**Figure 2:**
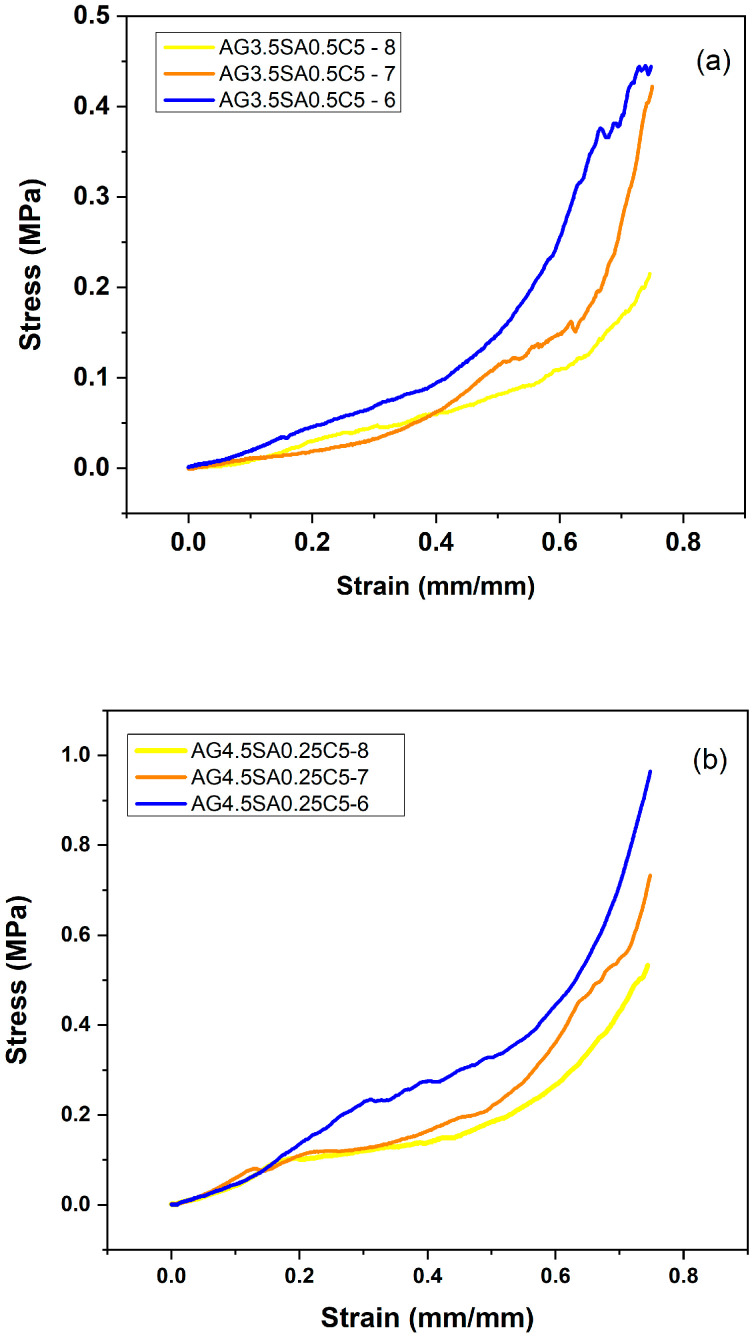

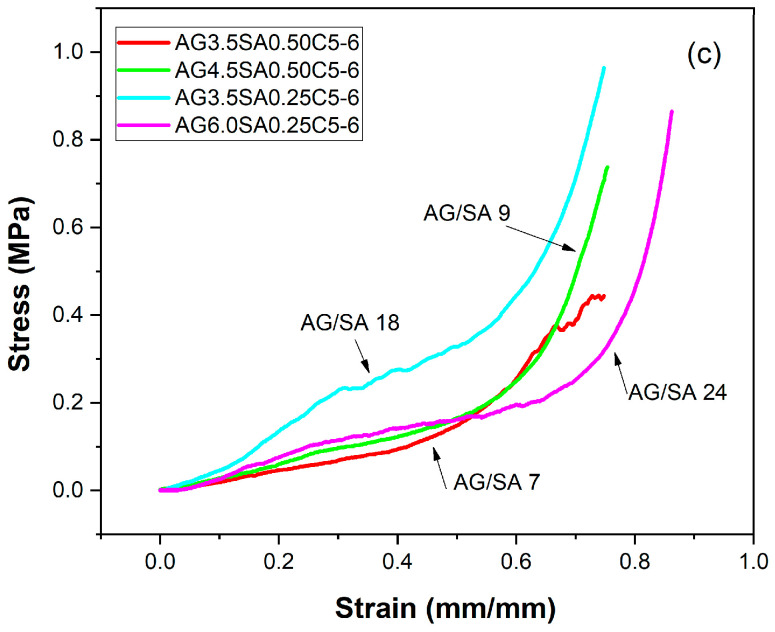
Stress–strain curves of aerogels based on ball clay with the incorporation of polysaccharides: (**a**,**b**) effect of pH and (**c**) effect of the ratio of AG/SA.

**Figure 3:**
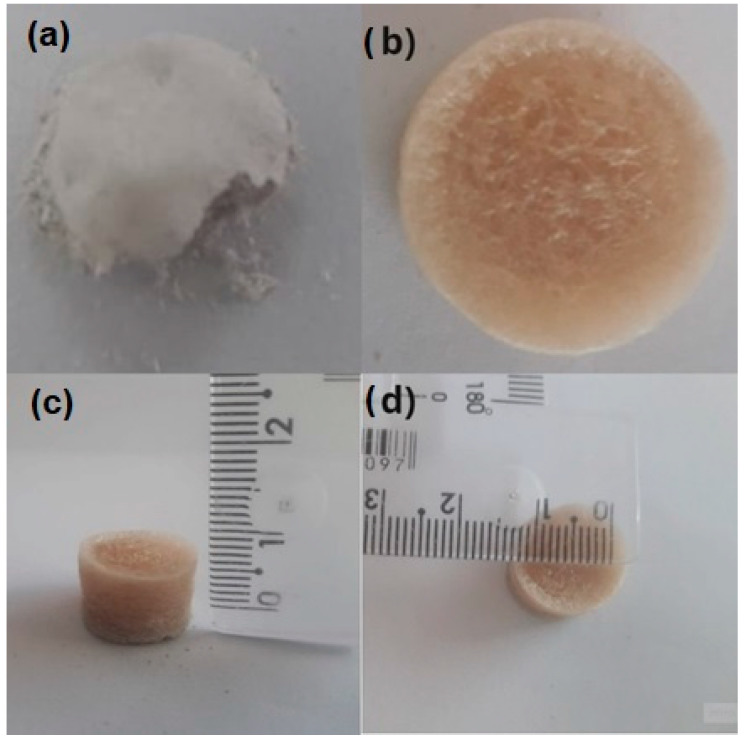
Photographs of (**a**) sample C-6 (aerogel produced from pure clay as a reference), (**b**) sample AG4.5SA0.25C5-6, (**c**) height, and (**d**) diameter.

The fragile mechanical resistance of aerogels is considered a problem [27], especially for industrial application as a thermal insulation material [28]. Figure 2 illustrates the stress–strain curves of the aerogels under different conditions, including pH (Figure 2a,b) and the AG/SA ratio (Figure 2c). In both batches with constant AG/SA ratio, the aerogels have superior mechanical properties at low pH values. With regards to the effect of the AG/SA ratio, Figure 2c and Table 2 suggest an increase in mechanical properties as the AG/SA ratio increased (7, 9, and 18), indicated by an increase in the Young’s modulus and a decrease in AG/SA 24. This phenomenon may be related to the lower viscosity of angico gum compared with alginate [19]. According to a previous study [29], the stress increased in all samples and the deformation increased slowly at the beginning and then more rapidly. This behavior is typical of a spongy material as observed in Figure 3b, which shows that the material initially has an elastic domain with a reversible deformation, the slope of the curve corresponds to Young’s modulus, and then there is a plastic region, where the fibrillar structure deforms irreversibly with increased stress [30]. Aerogels consisting of pure clay (Figure 3a) (produced as a reference without polysaccharides), showed very low mechanical strength and did not support any stress, evidencing the requirement for the incorporation of some polymeric material for structural strengthening [31].

The aerogels were deformed to around 80% of their initial length without breaking. Table 2 shows the apparent density, compressive strength, specific modulus (calculated by a division between compression modulus and density), and Young’s modulus of elasticity of the aerogels. The density is compatible with previous studies performed with aerogels of mixed matrices of chitosan/polybenzoxazine that presented densities between 0.051 and 0.073 g/cm^3^ [32] and aerogels of palygorskite/wood fiber (0.033–0.072 g/cm^3^) [29]. There was a slight decrease in density with varying the pH from 8 to 7, indicating that the addition of acid did not shrink the structure of the material [10]. Both the compressive strength and Young’s modulus of elasticity for the aerogels showed an increase associated with a decrease in the pH. The compressive strength of AG4.5SA0.25C-8 increased from 0.53 to 0.96 MPa and the Young’s modulus of elasticity of increased from 0.505 to 0.934 MPa when the pH decreased from 8 to 6, respectively. The specific modulus increased 14.2 times, going from 5.64 to 80 MPa. This result is similar to aerogels of crosslinked alginate with calcium chloride [22]. The pH decrease contributed to an improvement in the chemical interaction between polysaccharides and between polysaccharides and clay, providing the formation of a more compact three-dimensional network [33]. At low pH values, there is a change in the characteristics of polysaccharides, making them more physiologically positive and facilitating interaction with clay, which has an anionic character as shown in Table S1 in the Supplementary Data. This change in the properties of polysaccharides can occur due to the protonation of functional physiological groups, such as carboxylate groups, causing them to become positive. The interaction between the opposite charges of clay and polysaccharides can occur through electrostatic bonds or other intermolecular forces. Previous studies show that alginate is crosslinked at low pH values, which can result in a thicker alginate and clay solution [10]. The pH adjustment did not cause shrinkage in aerogels, which is considered better than the crosslinking normally used [10]. In this study, when maintaining the pH at 6 and increasing the percentage of AG to 33.5% there was an increase in the compressive strength of 0.96 ± 0.12 MPa to 1.44 ± 0.12 MPa. However, with a 100% increase in the percentage of the concentration of SA, the compression modulus decreased from 0.96 ± 0.12 to 0.74 ± 0.50 MPa. Comparing the specific modules with the increase in the concentration of polysaccharides indicated that a 33.5% increase in AG led to a twofold higher value than with the 100% increase in the alginate concentration. A recent study [34] described that with a fourfold increase in the percentage of cationic amylopectin in a montmorillonite clay aerogel of 2.1% by weight, the compression modulus of aerogels increased from 0.15 to 0.51 MPa.

### 3.2. Morphological Structure of Aerogels with pH Variation

**Figure 4:**
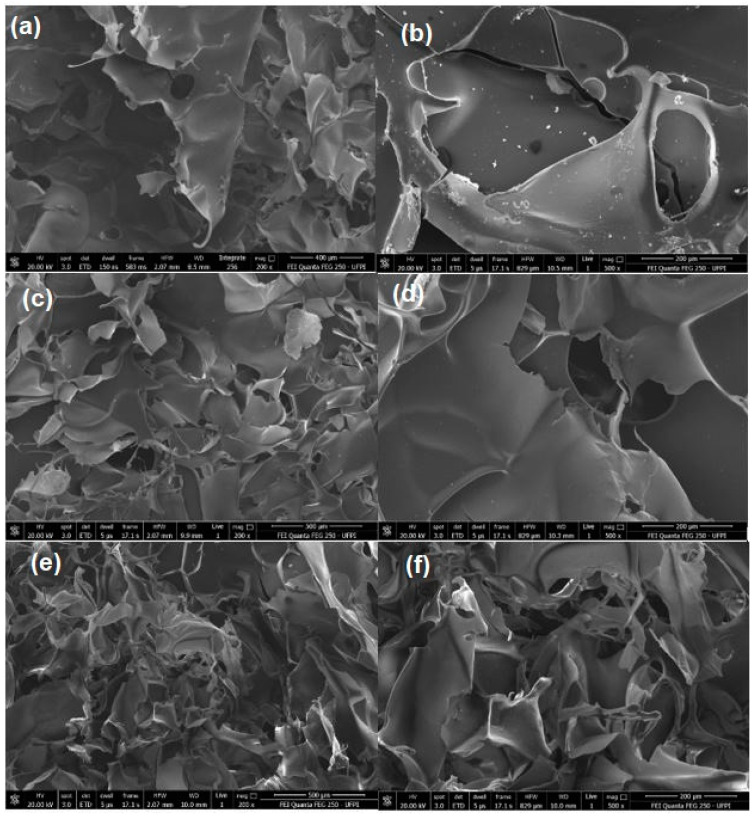
Scanning electron microscopy images of AG4.5SA0.25C5 hybrid aerogels with pH variation: (**a**,**b**) aerogel at pH 8, (**c**,**d**) aerogel at pH 7, and (**e**,**f**) aerogel at pH 6.

The morphological characteristics of the aerogels with variations in the pH are presented in Figure 4. The freeze-drying process resulted in aerogels in a 3D network structure and formation of interleaved networks in 2D leaf form [35]. Aerogels exhibit a porous structure and an increase in porosity density that is associated with a decrease in pH from 8 to 6. Table S2 presents the porosity results defined as the volumetric fraction of the voids. The magnification (Figure 4f) shows that the aerogel at pH 6 has a well-ordered formation that is compact and homogeneous in sheet form, making the aerogel more resistant to compression, as verified in the mechanical test presented in Figure 2.

### 3.3. Analysis of Crystalline Phases and Microstructure

**Figure 5:**
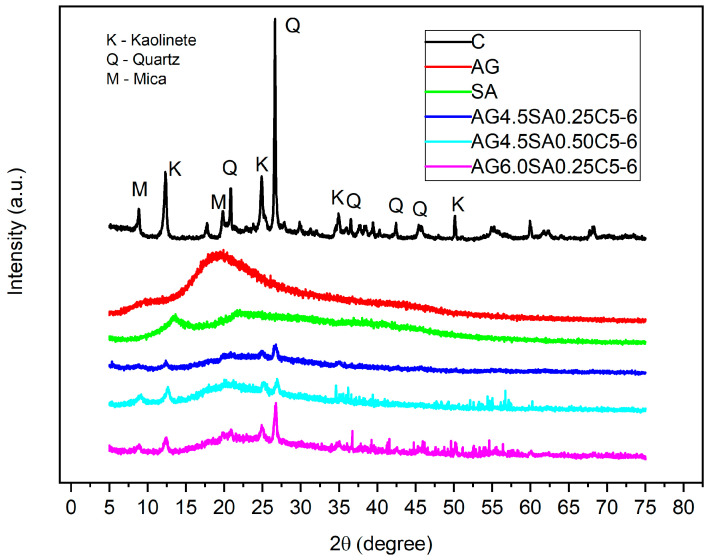
XRD patterns of ball clay, angico gum, alginate, and aerogels with variation in the mass of polysaccharides and at pH 6.

The crystalline phases of the materials were determined by X-ray diffraction (XRD). The results of mechanical tests and SEM showed a better result for aerogel samples at pH 6. The diffractions referring to aerogels at pH 8 and 7 are not presented. The diffractograms of polysaccharides and aerogels at pH 6 are represented in Figure 5. As was already reported for the gums, the AG diffractogram showed the characteristics of an amorphous material with a wide diffraction peak at 2θ = 19.5° and the SA was characteristic of a semi-crystalline material [36,37]. The SA diffractogram has an intense diffraction peak at 2θ = 13.36° that is related to a long-range chain order and occurs due to hydrogen bonds and polar intra- and intermolecular interactions [38]. The main diffraction peaks of clay (Figure 5), which refer to mica 2θ = 8.86, kaolinite 2θ = 12.28°, and quartz 2θ = 26.64°, are present in the aerogels without change in terms of the specific position; however, lamellar intercalation of the polysaccharides does not occur in the kaolinite. This can be verified using the Bragg formula (λ = 2d sin θ) since the d001 value for the most intense diffraction peak of kaolinite remained unchanged in the aerogels, with a basal spacing of 0.71 nm [39]. The EDS analyses (Figure S1) and Analysis of chemical characteristics by X-ray fluorescence spectrometry (Table S3) show results compatible with the chemical composition of kaolinite (Al_2_Si_2_O_5_ (OH)_4_), quartz (SiO_2_), and mica (KAl_2_Si_3_AlO_10_ (OH)_2_). The characteristic morphology of clay with high kaolinite content has its presentation in the form of crystals and in the form of lamella with irregular edges as shown in Figure S2. The graphs show peaks corresponding to the major constituents of the clay structure, including silicon (Si), aluminum (Al) with close intensities, potassium (K), and oxygen (O), along with other elements. In the aerogels, the reflections have lower intensity, indicating that the incorporation of polysaccharides in the aerogels was satisfactory. The relative crystallinity degrees are represented in Table 3. Even with the amorphous pattern of angico gums [40], the degree of crystallinity did not decrease with an increase in AG concentration from 4.5 to 6; this may be related to the nucleation effect, probably induced by the quantity of particles or particle size that are incorporated into the aerogel matrix [41]. 

### 3.4. Chemical Structure

**Figure 6:**
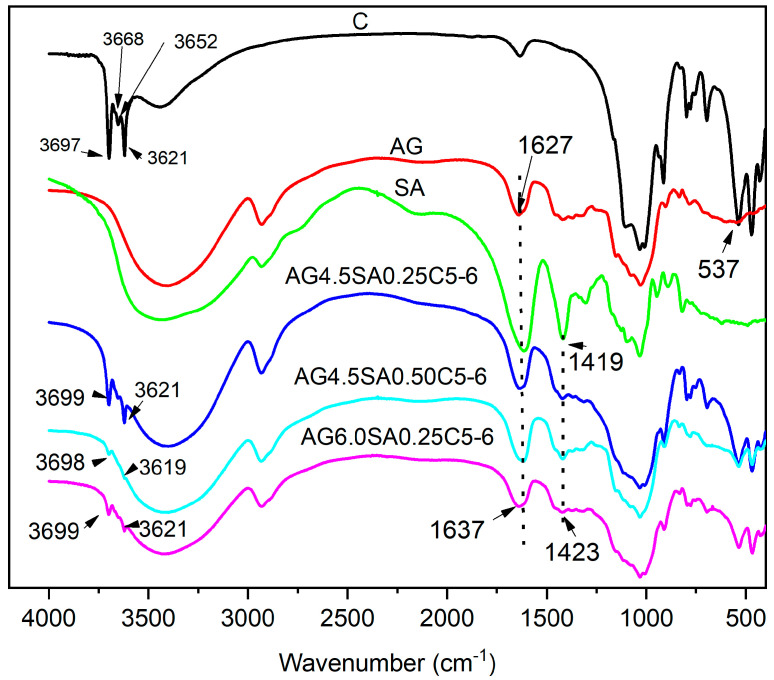
FTIR spectra of clay, AG, SA, and aerogels at pH 6.

In Figure 6, the FTIR spectra of AG, SA, and aerogels at pH 6 show absorption bands between 4000 and 400 cm^−1^ that are characteristic of polysaccharides. The bands at 2918, 1406, and 1018 cm^−1^ were assigned to -CH_2_ asymmetric stretch, -CH_2_ scissoring vibration, and C-O-C asymmetry stretch in the pyranose ring, respectively. The appearance of a wide and extensive band centered at 3437 cm^−1^ in the spectrum of the aerogels can be attributed to vibrations of O-H elongation due to the inter- and intramolecular interaction of the H binding of polymeric compounds [42]. In the FTIR spectrum of clay, the absorption bands in the region between 3700 and 3600 cm^−1^ correspond to the elongation of surface hydroxyl. The absorption band at 3697 cm^−1^ is associated with the external grouping of O-H located in the octahedral surface sheet of kaolinite; the absorption band at 3620 cm^−1^ is assigned to the inner O-H group of the tetrahedral plane of the laminar structure of kaolinite [43], which can interact strongly with polysaccharides by hydrogen bonding [44]. The mineralogical absorption bands at 1114 cm^−1^ and 1030 cm^−1^ refer to the Si-O stretch, whereas the band at 912 cm^−1^ refers to Al-OH flexion [24]. In aerogels, the main clay absorption bands are displaced longer wavelengths at the peak at 3697 cm^−1^ of the clay, with possible interactions between the polysaccharides and the functional groups of kaolinite. The FTIR results show that the asymmetric and symmetric COO- and OH- groups shifted to 1627 and 1419 cm^−1^ and proved a strong interaction between alginate and clay. A slight shift in the peak of alginate from around 1419 cm^−1^ to 1423 cm^−1^ can also be seen in the aerogel. In the aerogel FTIR spectrum with the same composition and pH value (8, 7 and 6) shown in Figure S3, it can be observed that the main peaks of ball clay (3697–3621 cm^−1^) do not show significant displacement. A slight change in the intensity of these peaks is observed at pH 8. The broad band between 3200 and 3500 cm^−1^ shifted to longer wavelengths with a decrease in the pH. In addition, the evolution of a new peak at around 1114 cm^−1^ at pH 7 was observed, which may be related to the Si-O stretching of kaolinite [45]. In all aerogels, the peak at 536 may be associated with the Al-O fold of the clay that may be displaced, suggesting a hydrogen bond between the polysaccharides and the clay [25]. The intense bands in the spectra of AG and SA are characteristic of the natural polysaccharides at 2918 cm^−1^, 1406 cm^−1^, and 1026 cm^−1^ that correspond to the vibrations of the asymmetric stretch of -CH_2_, vibration scissors of -CH_2_, and asymmetric stretch of C-O-C, respectively [46,47]. In general, the occurrence of displacements in the characteristic bands and changes in the intensity of the peaks present in the FTIR spectrum indicate the existence of flexible molecules between the biopolymers and the clay.

### 3.5. Thermal Stability 

**Figure 7:**
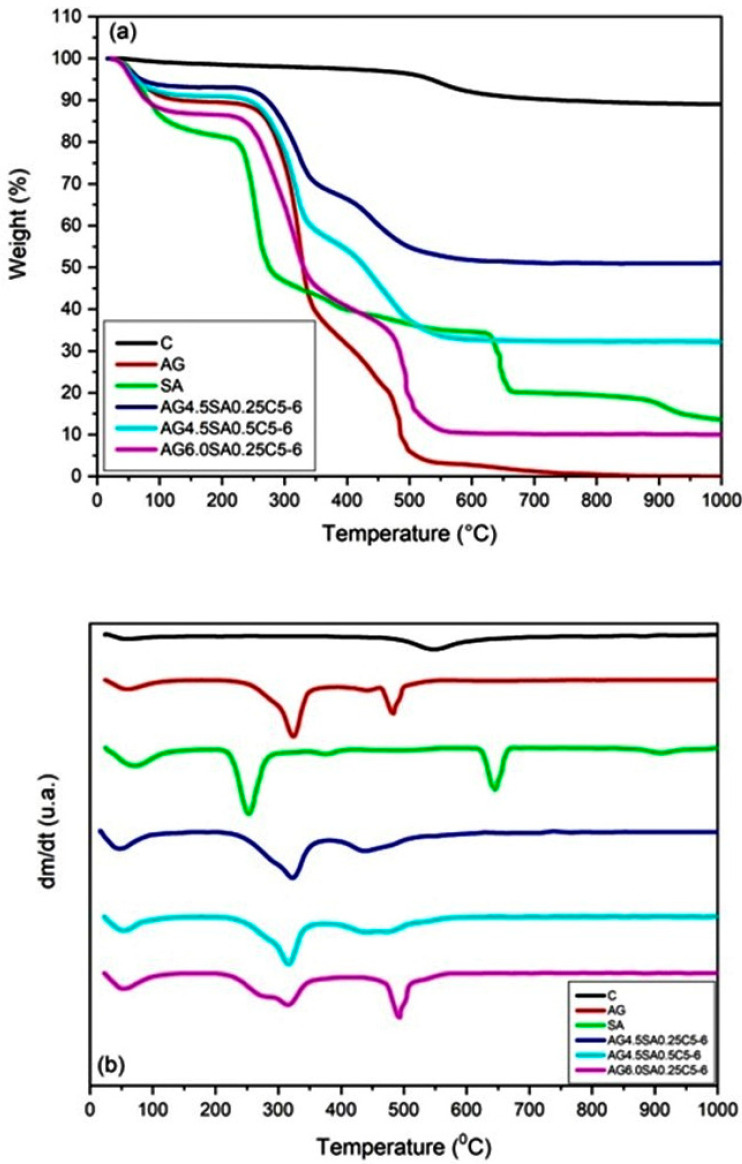
Thermogravimetric curves of clay, angico gum, alginate, and aerogels with variation in the polysaccharide mass and at pH 6: (**a**) TGA curves, (**b**) DTG curves.

The thermogravimetric curves in a non-oxidative atmosphere are represented in Figure 7, and the results are summarized in Table 4. The thermal behavior of the clay is similar to that reported in previous studies [15,18]. Figure 7a presents the TGA curve of clay, which presents two events. The first initially at 45 °C correspond to water loss. The second peak with has the highest mass loss in the region from 500 to 600 °C and is associated with the dehydroxylation of clay minerals and formation of metakaolinite [18]. The clay presented a mass loss of approximately 9% in the total heating process. Previous studies show that a mass loss close to 13% for ball clay confers better plasticity and refractoriness for the clay, a relevant factor for technological application [48]. The TGA curves of both AG and SA have three regions of mass loss. The first mass loss at temperatures below 100 °C was due to the loss of water and ethanol that were not eliminated in the drying process. The second and third mass loss regions correspond to the decomposition of the polysaccharides that occurs in two stages [49]. The TGA curves of the aerogels also show three regions with mass loss. The first is between 25 and 100 °C, which may be associated with absorbed moisture, indicating an adsorption of water molecules by the aerogel, a result similar to that described in previous studies [32]. The second and third mass losses in the aerogels are due to the degradation of the polysaccharides. The AG DTG peaks at 320 and 480 °C are also seen in aerogels, but with displacement of the peak at 480 to 490 °C in aerogels with higher concentrations of AG (Figure 3b). This temperature range displacement, when compared with the initial materials, indicates that there is interaction between the components. The loss peaks above 500 °C seen in samples C and SA were not observed in the aerogel degradation curves. In addition, a decrease in decomposition temperature in the aerogels with increased concentrations of polysaccharides can be verified. The increase in AG also showed a lower decomposition temperature than for SA.

**Figure 8:**
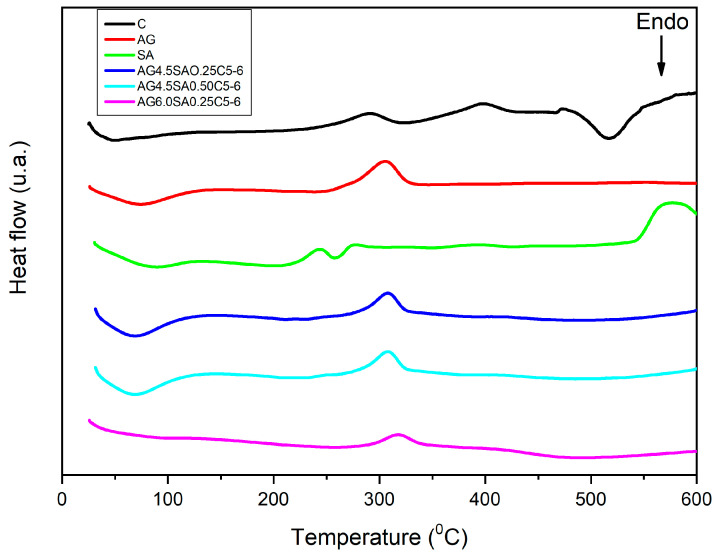
DSC thermogram of ball clay, angico gum, alginate, and aerogels.

The DSC curves are provided in Figure 8. The enthalpies calculated from the DSC data of different samples are in Table S4. The clay presents an obvious endothermic peak close to 517 °C, which may be the endothermic degradation of some type of organic material or the evaporation or fusion of this material. The AG DSC curve shows only two events, one endothermic, possibly representing water evaporation, and one exothermic at 306 °C, which is very characteristic of aerogels but with a displacement to 317 °C in aerogels with higher concentrations of AG. The SA curve is very different from the others as it has water evaporation and presents a composition of exothermic peaks with an endothermic peak in the temperature range 200 to 300 °C, as well as a characteristic exothermic peak in the range 540 to 600 °C. Aerogels with higher concentrations of AG did not show apparent loss of water. In addition, the crystalline state of the aerogels can be identified from the existence of endothermic peaks in the region of the melting point in the clay and SA curves that are not observed in the aerogels [47].

## 4. Conclusions

Aerogels based on ball clay and polysaccharides were produced through a physical process of mixing and drying by lyophilization, without the necessity of a chemical crosslinking agent. The mechanical tests prove the spongy structure of the aerogels, with an elastic deformation, a low density (0.012–0.097 g/cm^3^), and compressive strength of 1.4 MPa. A reduction in pH from 8 to 6 improved the mechanical properties without affecting the density of the aerogels, raising the specific modulus from 5.64 MPa to 80 MPa. XRD diffractograms showed that ball clay is comprised of kaolinite with basal spacing d_001_ = 0.71 nm, which makes it promising for aerogel production with the incorporation of polymeric components such as angico gum and alginate. The detection of displacements in the peaks of DTG suggest the existence of interaction between the polysaccharides and the clay, indicating that the ball clay has high thermal stability. The results of EDS and analysis of the chemical characteristics of ball clay by X-ray fluorescence spectrometry confirm the presence of alumina and silica, which results in a considerable resistance to temperature. These results open new possibilities for studies directed towards the application of this composite for thermal insulation. 

## Figures and Tables

**Table 1:** Measurements of pH and mass composition of ball clay and polysaccharides for aerogel preparation.

Mass Composition for Every 100 mL of Water					
Samples	pH	Angico Gum—AG (g)	Sodium Alginate—SA (g)	Ball Clay—C (g)	Distilled Water (mL)
C-6	6	0	0	5	100
AG3.5SA0.5C5-8	8	3.5	0.5	5	100
AG3.5SA0.5C5-7	7	3.5	0.5	5	100
AG3.5SA0.5C5-6	6	3.5	0.5	5	100
AG4.5SA0.25C5-8	8	4.5	0.25	5	100
AG4.5SA0.25C5-7	7	4.5	0.25	5	100
AG4.5SA0.25C5-6	6	4.5	0.25	5	100
AG4.5SA0.50C5-6	6	4.5	0.50	5	100
AG6.0SA0.25C5-6	6	6.0	0.25	5	100

**Table 2:** Mechanical properties of the aerogels.

Samples	AG/SA Ratio	Apparent Density (g/cm^3^)	Compressive Strength (MPa)	Specific Moduli (MPa cm^3^/g)	Young’s Modulus (MPa)
AG3.5SA0.5C5-8	7	0.089 ± 0.08	0.55 ± 0.15	6.17	0.205 ± 0.002
AG3.5SA0.5C5-7	7	0.090 ± 0.02	0.42 ± 0.08	4.65	0.323 ± 0.005
AG3.5SA0.5C5-6	7	0.077 ± 0.05	0.79 ± 0.14	10.25	0.464 ± 0.006
AG4.5SA0.25C5-8	18	0.094 ± 0.01	0.53 ± 0.02	5.64	0.505 ± 0.005
AG4.5SA0.25C5-7	18	0.012 ± 0.12	0.73 ± 0.03	60.83	0.688 ± 0.008
AG4.5SA0.25C5-6	18	0.012 ± 0.15	0.96 ± 0.12	80.00	0.934 ± 0.009
AG4.5SA0.50C5-6	9	0.096 ± 0.06	0.74 ± 0.50	7.71	0.605 ± 0.020
AG6.0SA0.25C5-6	24	0.097 ± 0.08	1.44 ± 0.12	14.84	0.574 ± 0.060

**Table 3:** Relative crystallinity of clay, polysaccharides, and aerogels at pH 6. Equation (1) was used for the calculation. A curve was drawn in the XRD diffractogram separating the area of the peaks; the area above the curve corresponds to the crystalline portion and the area below corresponds to the amorphous portion.

	Clay	Angico Gum	Alginate	AG4.5SA0.25C5-6	AG4.5SA0.5C5-6	AG6.0SA0.25C5-6
Crystallinity (%)	47.69	10.76	20.89	10.98	21.77	21.38

**Table 4:** Data for TGA of ball clay, polysaccharides, and aerogels at pH 6 in different percentages of decomposition.

Samples	Decomposition Temperature				Residues (%)
	T_d 5%_ (°C)	T_d 20%_ (°C)	T_d 30%_ (°C)	T_d 40%_ (°C)	
C	533	-	-	-	90
AG	56	287	307	320	0.5
SA	62	224	246	257	20
AG4.5SA0.25C5-6	70	314	351	451	51
AG4.5SA0.50C5-6	55	261	287	309	10
AG6.0SA0.25C5-6	64	294	315	341	32

## Data Availability

The data presented in this study are available only by the requesting them from the corresponding author due to the further work in progress.

## Supplementary Materials

The following supporting information can be downloaded at: https://www.mdpi.com/article/10.3390/polym15112412/s1, Table S1: The result from ζ potential gives natural clay treated at temperatures of 200, 400, and 600 °C; Table S2: Bulk density, theoretical density, and porosity defined as the volumetric fraction of voids; Figure S1: Energy dispersive spectroscopy (EDS) analysis of natural ball clay and ball clay treated at 200 °C; Figure S2: Scanning electron microscopy (SEM) images of natural ball clay samples and those treated at 200 °C; Table S3: Analysis of chemical characteristics by X-ray fluorescence spectrometry (XRF) of ball clay; Figure S3: FTIR spectra of samples of the same composition at different pH values (6, 7, and 8). Table S4: Enthalpy calculated from the DSC data for different samples: ball clay (C), angico gum (AG), sodium alginate (SA), and aerogels at pH 6.
