# Supplementary material for: Development of a New Clay-Based Aerogel Composite from Ball Clay from Piauí, Brazil and Polysaccharides
Source: Polymers (Basel). 2023 May 23;15(11):2412. doi: 10.3390/polym15112412 (PMC10255265; doi:10.3390/polym15112412)
Supplement: Supplementary file 1 [file polymers-15-02412-s001.zip › polymers-2301349-supplementary.pdf]

polymers

MDPI

Supplementary Material

Development of a New Clay-Based Aerogel Composite from *Ball Clay* from Piauí, Brazil and Polysaccharides

Wilton C. Lopes [1], Francisco M. Brito [1], Francisco E. Neto [1], Alyne R. Araújo [1], Rodolpho C. Leite [2], Vicente G. Freitas Viana [2], Edson C. Silva-Filho [3] and Durcilene A. Silva [1,*]

[1] Research Center on Biodiversity and Biotechnology, BIOTEC, Federal University of Delta of Parnaíba, UFD-Par, São Sebastião Avenue, Parnaíba 64202-020, PI, Brazil
[2] Postgraduate Program in Materials Engineering, Federal Institute of Piaui (IFPI), Campus Teresina Central, Teresina 64001-270, PI, Brazil
[3] LIMAV, Interdisciplinary Laboratory of Advanced Materials, Piauí Federal University, Teresina 64049-550, PI, Brazil
* Correspondence: durcileneas@gmail.com; Tel.: +55-86-33235433

The zeta potential was realized in the Chemistry laboratory of the Federal University of Piauí (UFPI) using an apparatus Zeta Sizer SZ 100 Horiba. For dispersive energy spectroscopy (EDS) analysis, an Oxford EDS detector was used. The chemical components present in ball Clay were analyzed quantitatively with the aid of an X-ray fluorescence spectrometer in Panalytical model Epsilon 3 XL equipment.

Table S1. The result from ζ Potential gives natural clay treated in temperatures of 200, 400, and 600 °C.

Samples	ζ Potential (mV)
Natural	- 69,3
200 °C	- 79,2
400 °C	-77,8
600 °C	- 68,6

The porosity defined as the volumetric fraction of voids V was calculated based on the work [25]. The calculation is done according to the equation (1)

$$V = \left(1 - \frac{\rho_{ap}}{\rho_{th}}\right) x\ 100\% \quad (1)$$

where ρ_{ap} is the apparent density of aerogel and ρ_{th} the theoretical density calculated according to the equation (2)

$$\rho_{th} = \frac{1}{\frac{w_{AG}}{\rho_{AG}} + \frac{w_{SA}}{\rho_{SA}} + \frac{w_C}{\rho_C}} \quad (2)$$

where W_{AG} , W_{SA} e W_C are the mass fractions in the dry aerogel of AG, SA, and ball clay (C), respectively. ρ_{AG}, ρ_{SA} e ρ_C densities of AG, SA, and ball clay, respectively. The density of the SA (0.4 g/cm3) was acquired from the supplier. The densities of AG (1.1 g/cm^3) and C (1.2 g/cm^3) were calculated using a glass pycnometer.

Table S2. Bulk density, theoretical density and porosity defined as the volumetric fraction of voids.

Samples	AG/SA ratio	Apparent density (g/cm^3)	Theoretical density (g/cm^3)	Porosity (%)
**AG3.5SA0.5C5-8**	7	0.089 ± 0.08	1.06	91.6
**AG3.5SA0.5C5-7**	7	0.090 ± 0.02	1.06	91.5
**AG3.5SA0.5C5-6**	7	0.077 ± 0.05	1.06	92.7
**AG4.5SA0.25C5-8**	18	0.094 ± 0.01	1.01	90.6
**AG4.5SA0.25C5-7**	18	0.012 ± 0.12	1.01	98.8
**AG4.5SA0.25C5-6**	18	0.012 ± 0.15	1.01	98.8
**AG4.5SA0.50C5-6**	9	0.096 ± 0.06	0.95	89.9
**AG6.0SA0.25C5-6**	24	0.097 ± 0.08	1.10	91.1

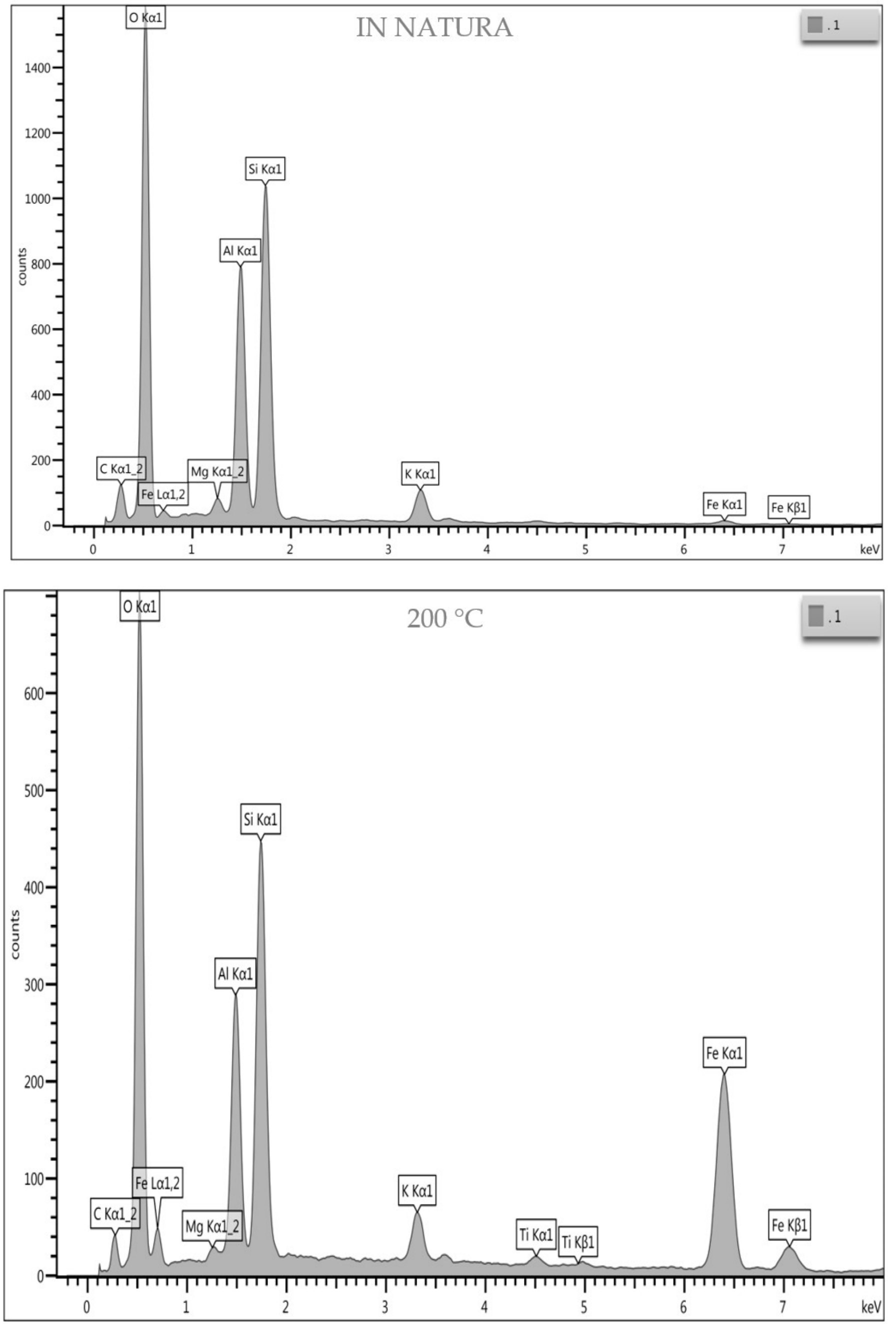

Figure S1. Spectroscopy analysis by energy dispersive (EDS) of ball clay in natural and 200 °C.

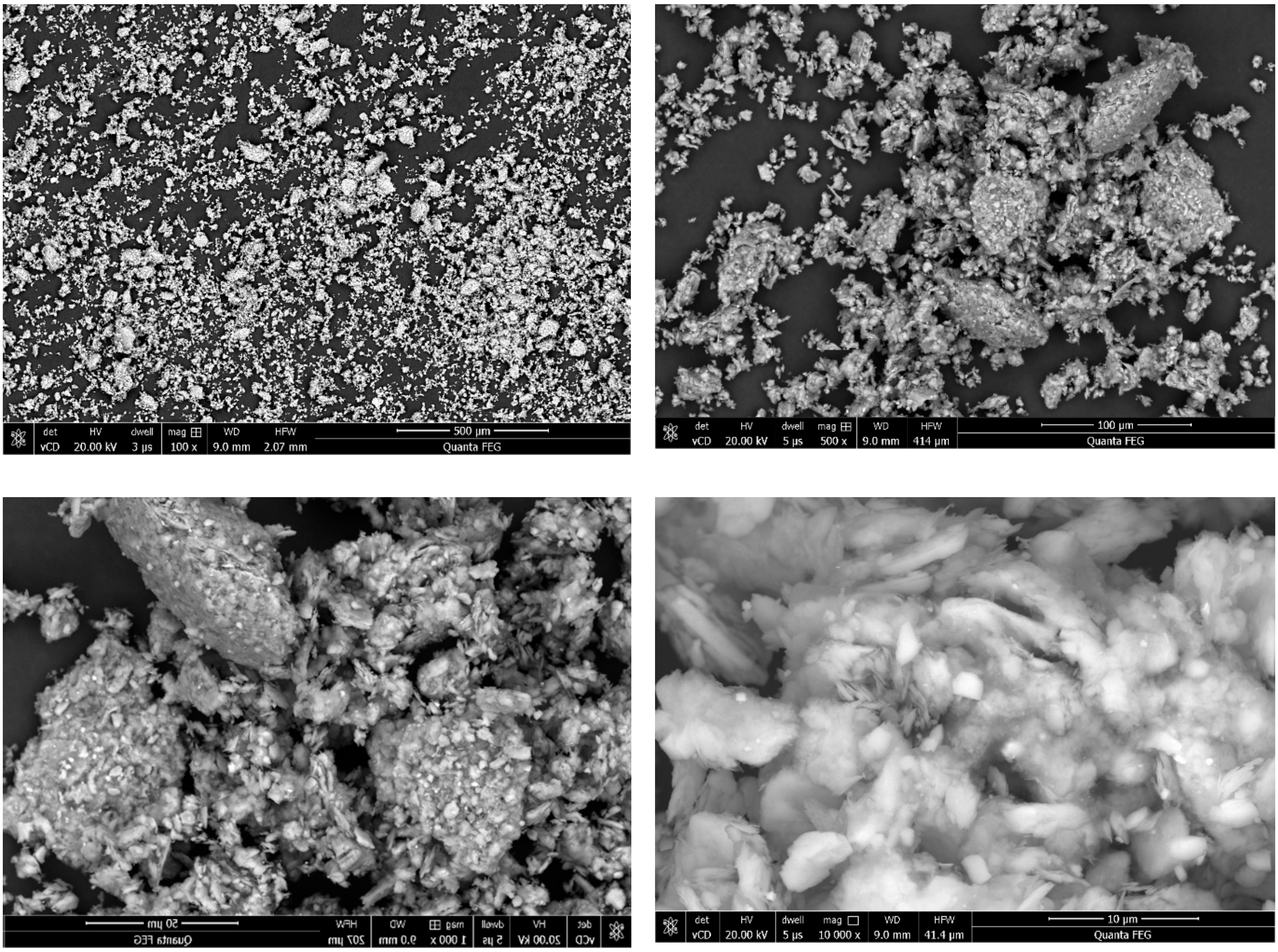

Figure S2. Scanning Electron Microscopy (SEM) images of ball clay samples treated at 200 °C.

Table S3. Analysis of chemical characteristics by X-ray fluorescence spectrometry (XRF) of ball clay.

**Chemical composition (wt%)**								
**PF**	**SiO_2**	**Fe_2O_3**	**Al_2O_3**	**TiO_2**	**CaO**	**MgO**	**Na_2O**	K_2O
15.37	58.83	4.27	29.71	1.32	0.11	0.83	0.58	4.21

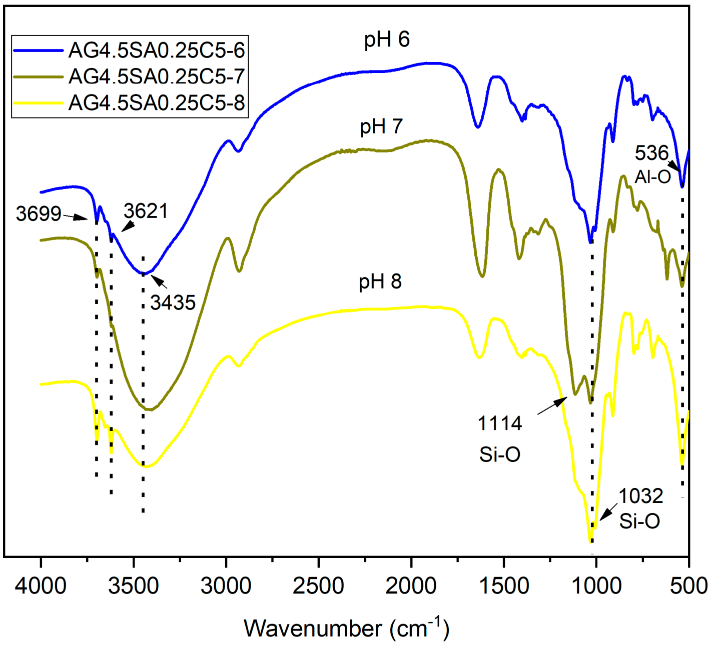

Figure S3. FTIR spectra of samples of the same composition at different pH (6, 7, and 8).

Table S4. Enthalpy calculated from the DSC data of different samples: ball clay (C), angico gum (AG), sodium alginate (SA) and aerogels with pH 6.

Samples	Peak Temperature (°C)	Δ*H* (mJ)	Δ*H* (J/g)
**C**	50	543.5	120.8
	291	-277.4	-61.6
	399	-330.4	-77.4
	516	885.7	196.8
**AG**	74	2870.0	478.2
	225	-17.4	-2.91
	306	-2990.0	-498.9
**SA**	85	1910.0	763.6
	244	-850.9	-340.4
	579	-1400.0	-558.4
**AG4.5SA0.25C5-6**	64	1010.0	459.2
	234	384.0	174.6
	313	-575.1	-261.4
	427	2890.0	1310.0
**AG4.5SA0.50C5-6**	68	1400.1	528.9
	211	233.5	97.3
	308	-759.5	-316.5
	485	278.4	115.9
**AG6.0SA0.25C5-6**	25	301.56	150.7
	140	-137.5	-68.7
	319	-361.2	-180.6
	473	74.82	37.4
